# Harmonic force spectroscopy measures load-dependent kinetics of individual human β-cardiac myosin molecules

**DOI:** 10.1038/ncomms8931

**Published:** 2015-08-04

**Authors:** Jongmin Sung, Suman Nag, Kim I. Mortensen, Christian L. Vestergaard, Shirley Sutton, Kathleen Ruppel, Henrik Flyvbjerg, James A. Spudich

**Affiliations:** 1 Department of Biochemistry, Stanford University School of Medicine, Stanford, California 94305, USA; 2 Department of Applied Physics, Stanford University, Stanford, California 94305, USA; 3 Department of Micro- and Nanotechnology, Technical University of Denmark, DK-2800 Kongens Lyngby, Denmark; 4 Department of Pediatrics (Cardiology), Stanford University School of Medicine, Stanford, California 94305, USA

## Abstract

Molecular motors are responsible for numerous cellular processes from cargo transport to heart contraction. Their interactions with other cellular components are often transient and exhibit kinetics that depend on load. Here, we measure such interactions using ‘harmonic force spectroscopy'. In this method, harmonic oscillation of the sample stage of a laser trap immediately, automatically and randomly applies sinusoidally varying loads to a single motor molecule interacting with a single track along which it moves. The experimental protocol and the data analysis are simple, fast and efficient. The protocol accumulates statistics fast enough to deliver single-molecule results from single-molecule experiments. We demonstrate the method's performance by measuring the force-dependent kinetics of individual human β-cardiac myosin molecules interacting with an actin filament at physiological ATP concentration. We show that a molecule's ADP release rate depends exponentially on the applied load, in qualitative agreement with cardiac muscle, which contracts with a velocity inversely proportional to external load.

## Abstract (web summary)

Single molecule methods for measuring load dependence are fundamental for molecular motor research. Here, Sung *et al*. introduce harmonic force spectroscopy, a method that randomly applies varying loads at high frequency, allowing the determination of load dependent parameters of human β-cardiac myosin at physiological ATP concentration.

## Graphical abstract

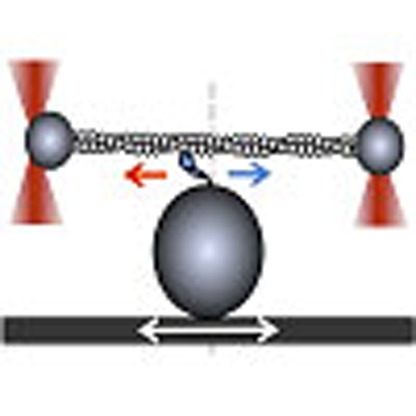

The human heart is the first functional organ to form during development, and it beats 90,000 times per day. It is a pertinent example of a loaded system and is powered by cardiac myosin motors^1^. Particularly interesting is the loaded behaviour of human β-cardiac myosin II, the primary isoform that generates contractile force in the left ventricle^2^. More than 300 single-missense mutations in this protein have been reported to cause hypertrophic cardiomyopathy (HCM)^3^, the leading cause of cardiac death in people under the age of 35 years. A fundamental characteristic of cardiac muscle contraction is its power output—the product of its force and velocity of contraction—at different loads^4^,^5^. While the power output of the heart is delicately poised and the force–velocity relation is pivotal in establishing it, purified human β-cardiac myosin has not had its load dependence characterized. Until recently, it was very difficult to obtain pure, functional human β-cardiac myosin for *in vitro* studies. This obstacle was removed with the expression of human β-cardiac myosin in a mouse C2C12 cell line system^2^,^6^,^7^. Another obstacle is the short-lived nature of motor interactions. Individual human β-cardiac myosins, for example, interact only transiently with actin at physiological adenosine triphosphate (ATP) concentrations (∼10 ms per ∼100 ms ATPase cycle, that is, with an ∼10% duty ratio)^1^,^6^. This requires fast tools that can detect the interaction and apply load with sufficient temporal resolution.

Several investigations of non-human skeletal or cardiac low-duty-ratio myosins have used either an ensemble of molecules in an *in vitro* motility assay at a saturating ATP concentration (≥2 mM)^8^,^9^ or a mini-ensemble in an optical trap assay at sub-saturating ATP concentrations^10^,^11^ to examine the collective load-dependent behaviour of myosin motors. These ensemble approaches are limited by the uncertainty about the actual number of interacting molecules in experiments. Furthermore, since they average over multiple molecules, they cannot resolve individuality in the load-dependent behaviour of the molecules. Finally, one needs to first understand the behaviour of a single molecule to understand the ensemble, because the latter could involve higher-order coupling effects among the ensemble of molecules^12^,^13^,^14^.

Study of the load dependence of single motors has required the development and improvement of various single-molecule force-spectroscopy techniques, including optical (laser) traps, magnetic traps and atomic force microscopy^15^,^16^,^17^. Typically, optical traps are used in a dual-beam filament–dumbbell configuration to probe a nonprocessively interacting motor anchored to a surface. In such an experiment, a constant load may be applied to the motor using either a force clamp or a position clamp^15^,^16^,^17^. Each of these clamped-force configurations applies force to a molecule after a binding event has been detected. Specifically, two implementations of these configurations have dominated the characterization of the load-dependent kinetics of myosin motors^18^,^19^,^20^,^21^,^22^,^23^,^24^: (i) a force-clamped actin-dumbbell configuration, in which a binding event was detected by measuring the binding-induced reduction of transmission of a sinusoidally oscillating signal from one trapped bead to the other through the actin filament strung out between them, followed by applying constant force^18^,^19^,^20^; (ii) a position-clamped actin-dumbbell configuration that applied dynamically changing loads to the motors near isometric force conditions^21^,^22^,^23^,^24^. Both configurations, however, require on-line detection of binding events followed by fast and robust feedback control of the instrument to apply a clamped force. This may give rise to a delay of several milliseconds between the time points of binding and loading, which is not ideal for transient molecular interactions^17^. Owing to this limited temporal resolution, most of these studies were carried out at sub-saturating ATP concentrations to lengthen the binding lifetime and may thus not reflect the actual loaded behaviour at physiological ATP concentrations. Alternatively, there have been other approaches for increased detection speed and sensitivity by oscillating either the trap or the stage^25^,^26^,^27^,^28^,^29^, and, recently, an ultrafast force-clamp optical trap with sub-millisecond temporal resolution was developed by Capitanio *et al.*^29^ To achieve that, the actin dumbbell was preloaded by oscillating it at a constant speed in a triangular wave-form, and binding events were detected as deviations in position of the bead from the trap.

Importantly, these clamped-force configurations all require complex instrumentation and careful operation for fast and robust feedback control, which might not be easily adaptable or extendable to a variety of systems. Taking an orthogonal approach, we here describe an easy-to-use, simple laser trap assay for measuring fast single-molecule load-dependent kinetics, applicable to molecular motors. Without a force clamp and without feedback control of the system, the instrument immediately, automatically and in random order, applies a range of different sinusoidal loads to a molecule that transiently interacts with a filament. These loads are generated directly by harmonic oscillations of the stage, in contrast to the established force-clamp configurations, where such oscillations are used solely to improve detection speed and sensitivity in the feedback control^29^. This simplicity makes ‘harmonic force spectroscopy' an easy assay with improved sampling efficiency as compared with existing methods, fast enough to achieve single-molecule results from single-molecule experiments.

To demonstrate the method, we subject human β-cardiac myosin subfragment-1 (S1, the motor domain of myosin) to this simplified assay. It yields directly the load dependence of the lifetime of the actin–myosin-bound state at saturating ATP concentration. The inverse of this lifetime is the adenosine diphosphate (ADP) release rate. Our results for this rate fit nicely to the Bell–Evans model for load-dependent rates of bond rupture, equation (16) in ref. 30. Also, our value for the ADP release rate at zero load agrees with the value obtained from ensemble stopped-flow experiments. This demonstration provides the first characterization of the load dependence of the ADP release rate of human β-cardiac myosin, which should be important in understanding various forms of genetic heart disease.

## Results

### Protocol for harmonic force spectroscopy

**Figure 1:**
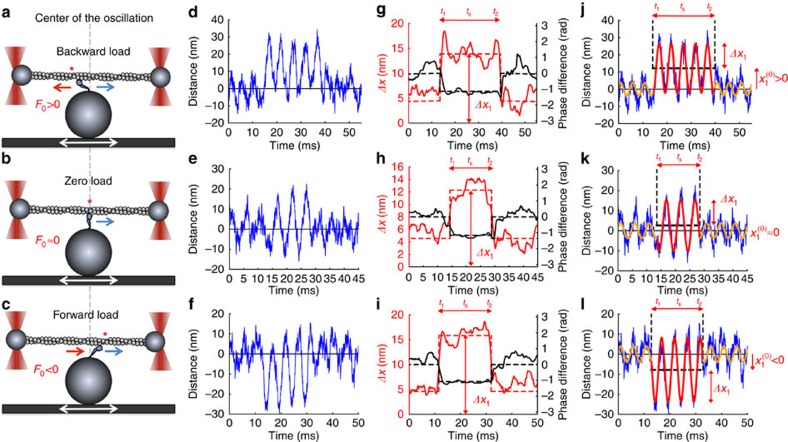
Harmonic force spectroscopy with human β-cardiac S1. (**a**–**c**) An actin dumbbell held by two fixed optical traps interacts with a β-cardiac S1 molecule that is surface attached to the top of a platform bead on a sinusoidally translating piezoelectric stage. Upon binding, the motor strokes towards the right (blue arrows). Depending on whether the binding occurs left of the centre (red asterisk in **a**), at the centre (red asterix on grey dashed line in **b**) or right of the centre (red asterisk in **c**), the motor experiences backward (**a**), near zero (**b**) or forward (**c**) mean load *F*_0_. (**d**–**f**) Three examples of time traces of the displacement *x*_1_ of a dumbbell bead in its trap. The other bead has coordinate *x*_2_ (not shown). Large sinusoidal oscillations caused by being attached to the stage by a motor are observed in the middle portion of the traces. Small oscillations before and after attachments are caused by drag from buffer moving past the unattached dumbbell. (**g**–**i**) Change in phase shift *ϕ*_1_(*t*) relative to the unbound state (black lines, dashed black lines showing its time average) and amplitude Δ*x*_1_(*t*) of oscillations (red lines, dashed red lines showing its time average obtained from longer stretches of unbound state times than shown) of bead positions in trap shown in **d**–**f** reveal binding and unbinding of motor (Methods). For each binding event, we determine the duration (*t*_s_=*t*_2_–*t*_1_) of the attached state, the mean load (*F*_0_) and the amplitude of load oscillations (Δ*F*) (Methods). (**j**–**l**) Data in **d**–**f** fitted with two harmonic functions, red for attached state and orange for unattached.

The following procedure quickly offers a range of loads to a single molecule, here a surface-anchored myosin S1 molecule^31^, enough to yield its load-dependent ADP release rate. We held the actin dumbbell over an S1 molecule with a conventional dual-beam laser trap, but moved the S1 harmonically along the filament with a piezoelectric stage (Fig. 1a–c), typically with ∼30–40 nm amplitude at 100–200 Hz (Fig. 1d–l; Methods). Figure 1a–c shows three examples of binding events. Figure 1a shows a case of myosin binding to the dumbbell while the stage and the myosin were undergoing an excursion to the left of their average position. The dumbbell then pulls left on the molecule, on average. With the motor oriented as shown, the dumbbell pulls in the opposite direction of the myosin stroke and hence applies a net backward force on the motor, that is, a positive mean load *F*_0_, where *F*_0_ is the oscillating load averaged over one or more cycles. Figure 1b shows a case of myosin binding to the dumbbell while the stage and the myosin were at their mean position. Consequently, the mean load on the myosin is zero. Figure 1c shows a case of myosin binding to the dumbbell while the stage and myosin were undergoing an excursion to the right of their average position. Consequently, the dumbbell pulls right on the motor, on average, in the same direction as the myosin stroke: there is a net forward force on the myosin, that is, a negative mean load *F*_0_. The load on the motor at a given instant is the sum of loads from the two laser traps at the same point in time. These loads were calculated from the displacements of the two beads in their respective traps by multiplication with the trap stiffness.

The advantages of this oscillation are twofold. First, it makes the attached states easy to detect and clock, as attachment makes the dumbbell oscillate with similar amplitude and phase as the stage (Fig. 1d–f, middle portion of the blue traces, which show the displacement of bead #1 of two in the dual-beam trap as a function of time). The red curves in Fig. 1g–i show the amplitude of oscillation calculated in a sliding time window of one period (wavelet transforms; Methods). The black curves show the phase of oscillations calculated in the same sliding windows. The actin–myosin-attached state is easily distinguished from the unattached state in two ways: the first is the increased amplitude of oscillation in the attached state as compared with the unattached state (Fig. 1g–i, red lines; Methods), and the second is a phase shift in the oscillation of the dumbbell upon attachment (Fig. 1g–i, black lines; Methods). This phase shift arises because, in the unattached state, the dumbbell oscillates in phase with the velocity of the stage, due to the drag force from the buffer, while in the attached state it oscillates in phase with the position of the stage (Methods). The second advantage is that the mean load experienced by the motor, while it pulls the trapped dumbbell with it through the cycles of the stage, depends on where in these cycles the motor attaches. It attaches at random anywhere in the cycle, so it experiences a range of backward- and forward-directed loads (Fig. 1a–c).

The red and orange curves in Fig. 1j–l show fits of harmonic functions to the bead's displacement in its trap while the myosin is attached and unattached, respectively, and illustrate that the bead moves as assumed in the wavelet analysis. Myosin bound to the actin dumbbell at *t*_1_ and released from the actin at *t*_2_, which gives the duration of attachment *t*_s_ (the strongly bound state time=*t*_2_−*t*_1_). Importantly, these experiments used saturating ATP (2 mM), so that the durations *t*_s_ of attachments should be a direct reflection of the rate of ADP release from myosin.

### Force modulates the ADP release rate of human β-cardiac S1

**Figure 2:**
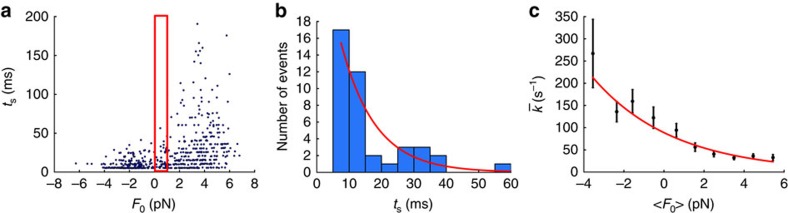
Load-dependent ADP release rate measured with 200-Hz stage oscillations. (**a**) Scatter plot of duration of binding (*t*_s_) versus load (*F*_0_) (*N*=388). (**b**) Histogram of durations *t*_s_ in the narrow range of loads in the red box in **a** (*N*=41). Histogram bins are 5 ms wide, that is, one period of the stage motion (Methods). The histogram is fitted by equation (5) using maximum likelihood estimation (MLE) to obtain 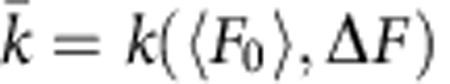. The histogram is consistent with an exponential distribution (red line). The characteristic time of this exponential is the inverse of the ADP release rate under the average load in the red box in **a**, 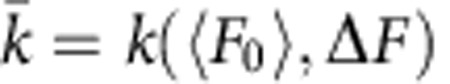. (**c**) ADP release rates depend exponentially on applied load. Black data points show ADP release rates 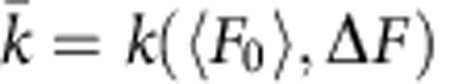 against mean load *F*_0_ for each of the 10 consecutive 1-pN bins. The individual error bars are calculated from the variance of the MLE in **b** as the inverse Fisher information for the parameter (Methods). The red curve is the fit of equation (1) to the rates, yielding *k*_0_=71±4 s^−1^, *δ*=1.01±0.09 nm, corresponding to *k*(0, Δ*F*)=89 s^−1^ on the red curve.

While attached to the trapped dumbbell, the motor experiences a harmonically oscillating load with amplitude Δ*F* and mean load *F*_0_ (Methods). Figure 2a shows a scatter plot of *t*_s_ versus *F*_0_. Each point denotes a single binding event such as those shown in Fig. 1. Importantly, all events in a single experiment, such as that shown in Fig. 2a, were recorded from a single human β-cardiac S1 molecule. The distribution of *t*_s_ changes significantly with *F*_0_: myosin detaches faster under forward load (*F*_0_<0) and slower under backward load (*F*_0_>0). We determined its load-dependent rate of detachment by examining the values for *t*_s_ that were recorded with a similar mean load *F*_0_, for example, those falling in the 0–1-pN bin (red box) in Fig. 2a. Figure 2b shows a histogram of these *t*_s_-values within the red box in Fig. 2a. This histogram is fitted by a single exponential function (red curve), which indicates that detachment is a Poisson process. It should reflect the ADP release rate from the myosin head, as the experiment used saturating ATP. We partitioned all data shown in Fig. 2a into 10 such 1-pN bins and obtained 10 different ADP release rates (Fig. 2c, black points). These rates fit well to a single exponential function (Fig. 2c, red curve), as expected from the transition-state theory and the Bell–Evans model for bond rupture (Methods): the load-dependent ADP release rate under harmonically oscillating load is


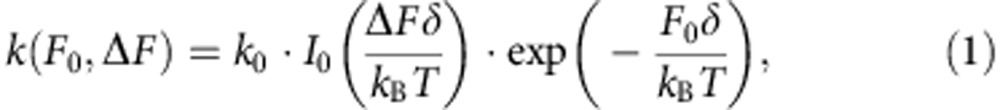


where the parameter *k*_0_=*k*(0, 0) is the rate at zero load, the parameter *δ* is the distance along the reaction pathway between the strongly bound-state and the transition state, *k*_B_*T* is the Boltzmann constant times the absolute temperature (the ‘Boltzmann energy') and *I*_0_ is the zeroth-order modified Bessel function of the first kind (Methods). This Bessel function factor accounts for the effect on the detachment rate from an oscillating load. In the limit of vanishing Δ*F*, *I*_0_(0)=1 and makes equation (1) take the form known for constant load *F*_0_, that is, 
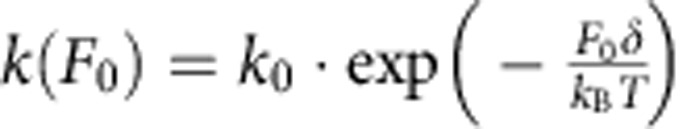
. Fitting of equation (1) to the data in Fig. 2c yielded *k*_0_=71±4 s^−1^ and *δ*=1.01±0.09 nm. Note that these values were obtained from data recorded for one particular molecule and hence characterize this molecule. Our protocol produced sufficient binding statistics (*N*=388) to characterize the load dependence of an individual motor molecule.

**Figure 3:**
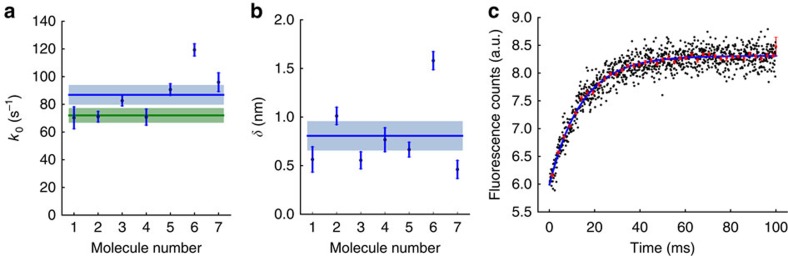
Individual parameter values (*k*_0_, *δ*) for seven independent measurements with six myosin molecules, #1 and #2 being independent studies of the same molecule. Each molecule is analysed as in Fig. 2. The stage is oscillated at 200 Hz, except for #1 (100 Hz). The number of attachment events for each molecule are, respectively, *N*=138, 388, 569, 174, 539, 950 and 229. Figures 1 and 2 use data for molecule #2. Blue points with error bars denote single-molecule results. The error bars are obtained from the theoretical covariance matrix (=inverse Fisher information matrix) of the single-molecule estimates for *k*_0_ and *δ* (Methods). (**a**) The load-free ADP release rates *k*_0_, corrected by 
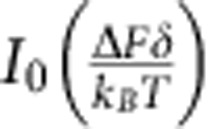
 to adjust for oscillation effects (Methods). Their weighted mean value, *k*_0_=87±7 s^−1^, is shown as a horizontal blue line with s.e.m. shown in light blue. If molecule #6 is treated as an outlier and dropped from our statistics, we find *k*_0_=80±7 s^−1^. The unloaded ADP release rate measured in stopped-flow experiments, *k*_0_=72±5 s^−1^, is shown as a horizontal green line with s.e.m. shown in light green. (**b**) The distance *δ* from the strongly bound state of each myosin to its transition state towards ADP release, measured along the reaction path. Their weighted mean value, *δ*=0.8±0.1 nm, is shown as a horizontal blue line with s.e.m. shown in light blue. If molecule #6 is treated as an outlier and dropped from our statistics, we find *δ*=0.7±0.1 nm. (**c**) Time trace of fluorescence changes (black points) from a stopped-flow experiment that measures the unloaded ADP release rate. The exponential fit (blue line) to binned data (red points) has a characteristic time of ∼14 ms (Methods). Such independent tests from five different preparations yield the release rate *k*_0_=72±5 s^−1^ (mean±s.e.m., *N*=5).

Figure 3 shows results from seven experiments with six different motors. They average to *k*_0_=87±7 s^−1^ (Fig. 3a, blue) and *δ*=0.8±0.1 nm (Fig. 3b) (mean±s.e.m., *N*=7). This average unloaded ADP release rate should reflect that observed in solution where no load is applied. To test this, we measured the ADP release rate by stopped-flow measurements in solution (Fig. 3c) and found it to be 72±5 s^−1^ (Fig. 3a, green) (mean±s.e.m., *N*=5), which agrees with 87±7 s^−1^. The *δ* of ∼1 nm reflects the conformational change in the molecule between the strongly bound ADP state and the transition state of the motor. Our results are similar to the values recently reported for porcine β-cardiac myosin using a position-clamped optical trap (*k*_0_=71 (−1.0/+0.8) s^−1^ and *δ*=0.97 (−0.014/+0.011) nm) by Greenberg *et al.*^24^ Note, however, that our load-dependent ADP release rate was obtained by averaging rates obtained for individual human β-cardiac myosin molecules with good statistics, rather than by combining data from multiple single molecules as done in ref. 24, presumably due to limited statistics^24^. The latter averaging must assume that the molecules averaged over are identical, while our method could determine whether they actually are identical or not. In our data, for example, molecule #6 seems to be an outlier as compared with the others, which shows the resolution of our method. If we leave out molecule #6 from our statistics, we find *k*_0_=80±7 s^−1^ and *δ*=0.7±0.1 nm, in better agreement with our stopped-flow result *k*_0_=72±5 s^−1^. Finally, note that motors #1 and #2 are the same molecule studied at two different frequencies of stage oscillation, 100 and 200 Hz, respectively. Data collection was efficient enough (*N*=138 and 388) to take measurements at both frequencies from the same molecule. The fitted values are consistent, which indicates that the oscillation used in our experiments does not affect the motor activity.

## Discussion

Harmonic force spectroscopy is beneficial in multiple aspects: simple instrumentation and operation, automatic and random sampling of load dependence, improved sampling efficiency, robust and fast detection of the signal and simple data analysis. Active force clamps or position clamps are powerful tools for characterizing load dependence of molecular complexes, but the necessary complicated apparatus for fast and robust detection of the response have been an obstacle to their general usage^18^,^21^,^29^. Harmonic force spectroscopy runs automatically, without any feedback control, by a simple sinusoidal oscillation of the stage, which produces load-dependent signals in a random manner, immediately upon binding. Harmonic oscillation of the stage allows improved sampling efficiency due to its continuous window for binding events in contrast to the previous oscillation-based methods in a square-wave form with feedback control, which has only a narrow window for applying the load^25^,^26^,^27^,^28^. The recent ultrafast force-clamp method developed by Capitanio *et al.*^29^ is elegant, but also uses only half of the oscillation cycle when the direction opposes the stroke. Our automatic binding-event detection algorithm uses not only the amplitude of oscillations to detect binding events, but also the phase change of oscillations that is caused by binding. This allows robust detection of the actual motor binding events in the presence of noise (Methods). Furthermore, the oscillation's amplitude and phase provide information about the compliance of the system (Methods). In addition, our data analysis is practically as simple as for clamped-force configurations, since one just needs the detachment kinetics under oscillating load provided in equation (1). In summary, this new method can resolve load-dependent transient interactions of low-duty-ratio molecular motors, and it is broadly applicable to other biological complexes, including protein–protein or protein–nucleic acid interactions.

Load-dependent ADP release has been previously reported for various types of myosin motors. For example, Veigel *et al.*^18^, using a force-clamp optical trap, have shown that two different myosin motors, smooth muscle myosin and myosin V^19^, exhibit similar load-dependent ADP release behaviour, despite their functional differences in cells. Laakso *et al.*^22^, using a position-clamp optical trap, have shown that myosin IB has load-dependent ADP release with extraordinarily strong load dependence (*δ*∼12), which supports its role as a molecular force sensor. Recently, Greenberg *et al.*^24^ reported a load-dependent ADP release of porcine β-cardiac myosin at saturating ATP concentration. The values reported by Greenberg *et al.* are similar to those in this study. Both their results and ours show relatively less load dependence (*δ*∼1) as compared with other, previously characterized myosin motors^18^,^19^,^22^. It was previously suggested that the β-cardiac myosin can generate more power under load due to the less-sensitive load dependence^24^. Overall, load-dependent ADP release seems to be a general strategy across various types of myosin motors (with a few exceptions^20^,^23^), although the degree of modulation appears to be specifically related to their roles in the cellular context^32^. It would be intriguing to further investigate the mechanism of the load-dependent ADP release by means of structural and/or mutational studies^20^.

The load-dependent ADP release rate of human β-cardiac myosin can explain the force–velocity relationship of contracting human cardiac muscle, conceptually and qualitatively. An approximate connection has been made earlier by identifying muscle shortening velocities with myosin stroke size divided by the duration of its strongly bound state time^33^, based on assumptions that individual motors independently interact with actin and the velocity is limited by the detachment of myosin from actin. We previously measured the stroke size of single β-cardiac myosin (∼6 nm) under low load^6^. Assuming that the stroke size is not load dependent^34^ and ADP release is the limiting step of muscle shortening velocity^35^, we can obtain a simple force–velocity relationship from our results: *V*(*F*)=*d*·*k*(*F*), where *V* is velocity, *d* is the stroke size and *k* is the ADP release rate. However, this simplified model might not fully explain the force–velocity curve of contracting muscle, and there could be other possible mechanisms to be considered. For example, the stroke size could also be load dependent^36^,^37^, and/or the stroke could be reversed^21^, slipped away^10^ or prematurely completed under high load^29^. Furthermore, the characteristics of single myosin molecules might not fully explain that of motors as an ensemble^12^,^13^,^14^. Particularly, Walcott *et al.*^12^, using a combination of simulations and theory, proposed that even internal forces generated between myosin molecules through actin can act as load and can affect ADP release rate and velocity. Further modelling studies would be valuable to make a more compelling connection between single-molecule and ensemble results. Nevertheless, the loaded ADP release behaviour of single molecules discovered in this study should play an important role in contracting muscle and improves our understanding of the Fenn effect^4^ and the force–velocity curve^5^ in human cardiac muscle.

Moreover, understanding such behaviour in human β-cardiac myosin will play an important role in deciphering the effects of HCM and dilated cardiomyopathy (DCM) causing mutations in the very same protein. In fact, Debold *et al.*^11^, using a force-clamp optical trap with a few mouse α-cardiac myosin heads, measured the force–velocity relationship for two HCM and two DCM causing myosin mutants. They showed that the force–velocity relationship is altered by the mutations, implying that the underlying loaded ADP release rates of myosin are affected. Separately, measures of isolated mouse cardiac myofilament velocity and power under loaded conditions were shown to be more sensitive for a particular HCM-causing myosin mutant^38^. Such changes are presumably again due to differentially loaded ADP release rates for the mutants as compared with wild type. It should be noted that the changes in kinetics and force due to HCM and DCM mutations are often very small (in the 10–50% range) but highly significant; thus, small differences between large animals, such as porcine, will be extremely important in the context of HCM and DCM, and it is critically important to use the very same protein as in this work. Our new method, with its simplicity and efficiency, should now be useful in defining such differences in the force–velocity curve and the power output of an individual single human cardiac actin–myosin system resulting from such mutations in human β-cardiac myosin and other sarcomeric proteins^1^,^3^. Understanding the molecular basis of the alterations caused by the mutations will be also useful in developing small molecules for new therapeutic approaches to mitigate the dysfunctions caused by genetic disease^1^,^39^. In summary, the simplicity and resolution of our method offers new opportunities to understand the effects of force in life and how such effects are altered in a diseased state.

## Methods

### Proteins, reagents and buffers

Human β-cardiac myosin subfragment S1 (ref. 31) was prepared as follows. A detailed description of the procedure is also given elsewhere^2^,^6^. Briefly, we designed the AdEasy Vector System (Qbiogene, Inc.) containing a short S1 version of MYH7 (residues 1–808), truncated after the MYL3- (ventricular essential light chain; ELC) binding site, followed by a 1 × GSG flexible linker and a green fluorescent protein (GFP) tag for surface anchoring. Replication-deficient recombinant adenoviruses were produced and amplified in HEK293 cells, and two different viral particles containing the S1 and ELC plasmid, respectively, were purified and concentrated. Mouse C2C12 myoblasts were cultured and co-infected with the S1 and ELC viruses. We used anti-FLAG resin to purify FLAG-tagged ELC that bound to S1, followed by ion-exchange chromatography on a 1-ml HiTrap Q HP column (GE Healthcare) to separate it from endogenous mouse skeletal myosin. After purification, the protein concentration was deduced from its GFP absorbance (molar extinction coefficient at 488 nm=55,000 M^−1^ cm^−1^). We further eliminated inactive or damaged myosin using a method termed ‘dead-heading' by sedimenting (at 100,000*g*) non-recyclable motors that remain rigour bound to actin filaments in the presence of 2 mM ATP concentration.

Actin was prepared from fresh chicken-breast skeletal muscle and further purified by recycling it from G- to the F-form^6^,^40^. It was subsequently biotinylated and fluorescently labelled with tetramethyl rhodamine (TMR)-phalloidin (Invitrogen)^6^,^41^. For optical trap measurements, biotin-labelled polystyrene beads (1 μm in diameter, Invitrogen) were labelled with streptavidin and TMR–BSA as previously described^6^,^41^. Glass coverslips were spin-coated with silica platform beads (1.5 μm in diameter) and coated with 0.2 % nitrocellulose in amyl acetate (Ernst Fullam).

All trap experiments were performed in assay buffer (AB) (25 mM imidazole (pH 7.5), 25 mM KCl, 4 mM MgCl_2_, 1 mM EGTA and 10 mM dithiothreitol (DTT)) at 23 °C. The final experimental solution consisted of AB with 1 mg ml^−1^ BSA (ABBSA), 2 mM ATP, an ATP regeneration system (100 μg ml^−1^ creatine phosphokinase and 1 mM phosphocreatine), TMR-phalloidin-labelled biotinylated actin filaments (∼1–2 nM), an oxygen-scavenging system (0.2 % glucose, 0.11 mg ml^−1^ glucose oxidase and 0.018 mg ml^−1^ catalase), streptavidin-coated polystyrene beads and 1 mM non-fluorescent phalloidin.

### Sample chamber preparation

A nitrocellulose-coated sample chamber, coated with 1.5-μm silica beads as platforms, was prepared as described^6^. Anti-GFP antibody (∼1–10 μg ml^−1^) (Millipore) was flowed through the chamber, followed by blocking the surface with BSA. Human β-cardiac S1 was then anchored to the antibody on the surface through its C terminus GFP tag. A typical concentration of ∼100 pM was used to ensure single-molecule binding events. The chamber was washed with ABBSA, filled with the final experimental solution and then sealed with vacuum grease.

### Harmonic force spectroscopy: experiment

The dual-beam laser trap set-up and the techniques used in the present study is described in detail elsewhere^42^. Briefly, we trapped two streptavidin-coated polystyrene beads with the two beams, and we carried out a trap calibration procedure to obtain the trap stiffness in pN nm^−1^ and the quadrant photodiode (QPD) position detector sensitivity in nm V^−1^ (see below). The typical trap stiffness of each trap in this study was ∼0.1 pN nm^−1^. After this calibration, an actin dumbbell was formed, tightly stretched and the taut actin dumbbell was gently moved into proximity of the platform bead where a myosin motor might be anchored. We oscillated the piezoelectric stage with amplitude 50 nm at 100 or 200 Hz until we found an event of binding on a platform bead. Typically in all of our experiments we had to explore ∼10–15 platform beads before we could observe any binding with myosin, suggesting that only a single molecule was binding when binding occurred. We then stopped oscillating the stage and checked for unidirectional stroking events from the motor to make sure that binding was indeed by an active motor. That confirmed, the stage was oscillated again with no interruption, and the positions of the trapped beads and the piezoelectric stage were recorded at 40 kHz sampling frequency until the motor became inactive.

### Calibration of the dual-beam laser trap set-up

After trapping a bead in each optical trap, we brought them close to the coverslip where measurements of motor function were performed. There we calibrated the experimental set-up following a two-step procedure. First, we calibrated the QPDs using the following raster scan procedure^43^. Briefly, we displaced the beads by 60-nm steps over an area of 600 × 600 nm using acousto-optic deflectors; we recorded the beads' displacement at each step, both with a video camera (in nm) and with the QPDs (in mV). This calibration allowed us to relate the QPD signal to physical displacement. For experimentally relevant displacements, the QPDs show linear response, and the QPD signals thus directly give us the beads' positions via a simple V-to-nm conversion factor for each coordinate and for each QPD. Second, we calibrated the traps using maximum likelihood fitting to the power spectrum of the recorded thermal motion of the beads in the traps^44^,^45^. We used the QPDs to record the positions of the beads for ∼25 s (10^6^ data points) and calculated the power spectra of the recorded time series using windowing (4,000 data points in each window) as described in section 12.7 of Press *et al.*^46^. We fitted each power spectrum with a sum of two Lorentzians while taking into account low-pass filtering by our anti-aliasing filter and aliasing of the filtered signal^47^. One Lorentzian accounts for low-frequency instrumental drift of the trap's centre; the other Lorentzian describes the thermal motion of the bead. The fitted parameters of this last Lorentzian are estimates of the trap's stiffness and the bead's drag coefficient *in situ*, near the surface where the calibration and the experiment were done^44^,^48^.

### Compliances

Compliance in the actin dumbbell is likely mainly due to rotation of beads combined with bending of the tangentially attached actin filament (see Fig. 5 in Dupuis *et al.*^49^), but potentially has also contributions from bead linkers (streptavidin–biotin interactions) and the motor linking the dumbbell to the stage (in S1, GFP and GFP antibody)^18^,^21^,^29^,^42^. In general, compliance makes the geometry of the attached dumbbell change elastically in response to the periodically varying force on it from the traps that pull on its beads, while one point on the dumbbell handle is forced to follow the stage closely. This periodic change in geometry means that the dumbbell beads do not move with the exact same velocity as the stage, but with lower amplitude and positive phase shift. Thus, the phase of the attached states shown in Fig. 1g–i is not a full *π*/2 behind the phase of the unattached state, but is less negative because of this positive phase shift due to compliance. The compliance of the dumbbell is also partly the reason why stage oscillations with a nominal 50-nm amplitude give rise to bead oscillations with amplitudes of only 12–16 nm in Fig. 1g–i. Another reason is that the actual stage amplitude is less than its nominal amplitude (for example, ∼35 nm rather than 50 nm at 200 Hz oscillation) at the relatively high frequencies we oscillate it with. This is so because the piezo stage has a resonance frequency around 500 Hz. If oscillated at this frequency, it may be damaged. As a safeguard against this, it has a built-in low-pass filter that attenuates the voltage driving the stage, and hence the amplitude of stage oscillations, as the resonance frequency is approached. The actual oscillation amplitude was measured from the sensor voltage of the piezo stage, which was pre-calibrated with a calibration bar. The difference in velocities between dumbbell beads and stage gives rise to a Stokes drag force on the beads from the buffer fluid, as the fluid moves with the same velocity as the stage. The load on the S1 from each dumbbell bead is the sum of the trapping force and the drag force on the bead. The trapping force we measure as the displacement of the bead in the calibrated trap. The drag force can be calculated (Supplementary Note 1) using the measured positions and velocities as input (Supplementary Fig. 1). It turns out to phase shift the load, but affects its amplitude only negligibly because the velocity, and hence the drag force, is phase shifted *π*/2 ahead of the trap force it adds to.

### Harmonic force spectroscopy: theory

The mean value *F*_0_ of the oscillating load on the myosin depends on where in the cycle of the periodic motion of the stage the myosin happens to attach to the dumbbell. *F*_0_ is minus the sum of the mean forces from the two traps on the dumbbell, which we measure. Thus, we measure the lifetime of the attached state of myosin for a range of measured *F*_0_-values. Figure 2a shows a scatter plot of this *F*_0_ versus lifetime. It is interpreted as follows: let *k*(*F*) denote the load-dependent rate of unbinding of myosin from actin. Let *F* be a function of time *t*. Then the ‘survival function' *P(t*_1_,*t*_2_) of the attached state—that is, the probability of remaining attached from time *t*_1_ of binding till a later time *t*_2_—evolves in time according to the quasi-stationary dynamics.


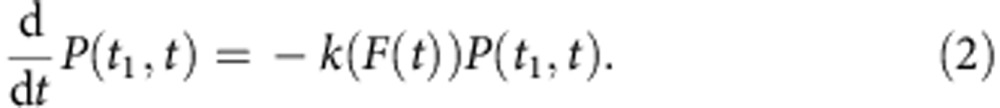


This dynamics is solved by


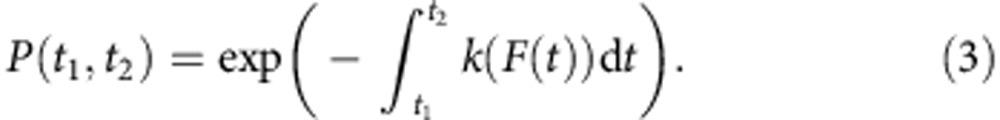


The probability density function (PDF) on the time axis for unbinding at a time *t*, given that binding took place at time *t*_1_, is equal to minus the right-hand side in equation (2). At constant load *F*, as in force-clamp spectroscopy, this PDF is a simple exponential that depends only on the difference *t*–*t*_1_. For general time-dependent *F*, this PDF is the more complicated function that results from inserting equation (3) in the right-hand side of equation (2). It depends explicitly on both *t* and *t*_1_. This complicates the data analysis. However, if *F* is a periodic function of *t*, and *t*_2_–*t*_1_=*n t*_drive_ is an integer number of periods *t*_drive_ of *F*, then


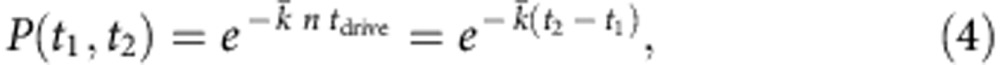


where 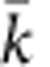 denotes the time average of *k*(*F*(*t*)) over a period. This is a simple exponential function of *n*. In our data analysis we use the discrete PDF describing the probability of unbinding in the period following *n* periods in the bound state, which is


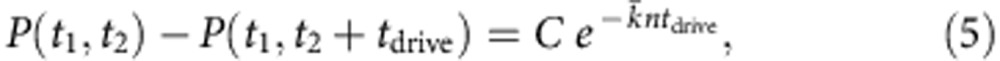


where 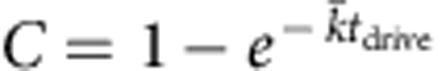. This also is a simple exponential function of *n*, known as the geometric distribution. It shows that ‘periodic force spectroscopy', as we call it, in its data analysis is as simple as force-clamp spectroscopy. Its use only requires that the period, *t*_drive_, is shorter than the timescales one wishes to resolve.

We achieve further simplifications by using a periodic load that is harmonic, in what we call ‘harmonic force spectroscopy',


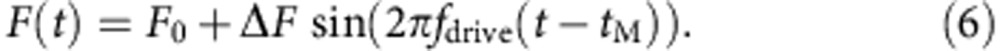


It has *f*_drive_=1/*t*_drive_ and *t*_M_ denoting the arbitrary phase of the oscillating stage. As Δ*F* differs little between binding events, *F*_0_ is the only quantity in this expression that really differs between binding events. Equation (5) therefore predicts that binding events that have (nearly) the same mean load *F*_0_ have attachment times that are exponentially distributed in time, when binned on the time axis in bins that last an integer number of periods of the stage motion, for example, one period. Figure 2b shows that this is, indeed, the case. In this manner, we determine 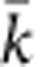 experimentally for a range of values for *F*_0_. Figure 2c shows a plot of these values. This plot describes a load-dependent ADP release rate that is convincingly described by an exponential dependence on the load *F*_0_. This observation points towards Arrhenius' equation,


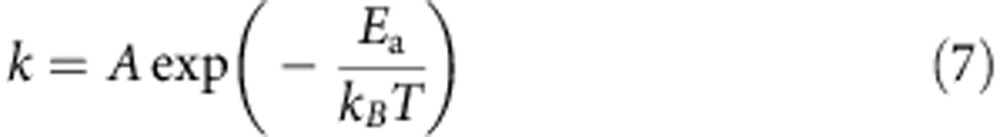


and the transition-state theory. In equation (7), *E*_a_ is the activation energy, the energy difference between the bound state and the transition state for detachment. If a constant external load *F* opposes the transition, *E*_a_ is increased by the amount of work done by the myosin against that load. With *δ* denoting the distance between the bound state and the transition state, as measured along the reaction pathway, the height of the energy barrier towards unbinding is increased from *E*_a_ to *E*_a_+*Fδ*. Consequently,


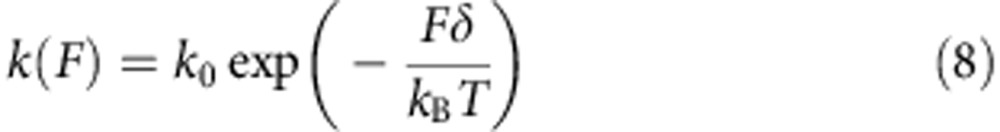


and hence, with our harmonic force in equation (6),


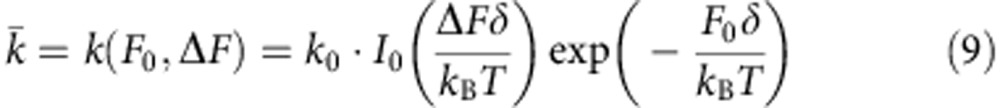


where *I*_0_ is the zeroth-order modified Bessel function of the first kind. Thus, we see that Arrhenius' equation explains the observed exponential dependence of 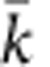 on the mean load *F*_0_. As *k*_B_*T* and Δ*F* are known, a fit of the last equation to data, using *k*_0_ and *δ* as fitting parameters, determines these two parameters of the load-dependent ADP release rate; see Fig. 2c.

### Detection of binding events

We detect events of myosin binding to the actin dumbbell by monitoring the displacements of the individual bead in its optical trap (Fig. 1d–f). When the myosin is not bound to the dumbbell, the dumbbell oscillates due to drag from the fluid in the sample chamber (Fig. 1d–f, left and right portions of the traces), which follows the stage motion. In this oscillation, the dumbbell position follows the fluid velocity closely and is therefore ahead of the stage position by almost *π*/2, see equation (7) in Tolić-Nørrelykke *et al.*^44^. When the myosin is bound to the actin dumbbell, the oscillation of the piezoelectric stage is transferred to the trapped beads, and the positions of the beads are now almost in phase with the oscillating position of the stage (Fig. 1d–f, centre portions of the traces)—compliance of the dumbbell allows the beads to get a little ahead of the stage position, dragged by the fluid in the sample chamber, but the net effects of binding are oscillations of the dumbbell with much larger amplitude, typically around a different mean position, and a phase shift by almost −*π*/2 relative to the phase of oscillations in the unbound state (Fig. 1g–i, black traces). Thus we have two criteria for selection of binding events: changes in amplitude (Fig. 1g–i, red traces) and changes in phase (Fig. 1g–i, black traces), such as the signals of AM and FM radio, respectively, except we ‘listen' to both simultaneously.

In practice, we analysed the data in time windows of 10,000 positions of each dumbbell bead, recorded at 40 kHz. In each such window, the time series of positions were wavelet transformed at the stage frequency (100 or 200 Hz) as follows. From each time series, two time series were formed by multiplying the bead's positions, respectively, with the sine function fitted to the stage positions, and with the cosine function obtained by phase-shifting this sine by *π*/2, after proper normalization of both. Sliding time averages over one stage period were formed from these two series. We denote these averages by *s*(*t*) and *c*(*t*), respectively. They are our wavelet-sine- and wavelet-cosine transforms. We then calculated the amplitude of oscillations in the signal as 
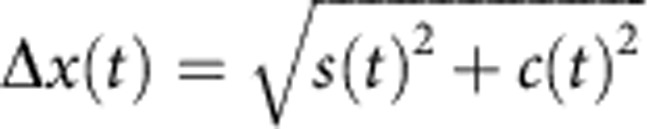
, and the phase of these oscillations as *ϕ*(*t*)=arctan(*c*(*t*)/*s*(*t*)). We detected potential binding events by recording time intervals in which the phase dropped below the phase of the unattached state by more than a threshold value and, simultaneously, the amplitude of oscillations passed above a threshold value (Fig. 1g–i). We typically demanded a drop in the phase *ϕ*(*t*) by >0.5 rad and a simultaneous increase in amplitude making Δ*x*(*t*)>10 nm for at least one full stage period for either bead. This was a first filter against false binding events caused by Brownian motion. Typically, beads would pass thresholds simultaneously, however.

For each potential binding event in each bead's trajectory, we averaged the amplitude Δ*x*(*t*) of oscillations (Fig. 1g–i, solid red lines) over the duration of attachment, starting half a period after binding and ending half a period before unbinding. The resulting average Δ*x* (Fig. 1g–i, dashed red lines) multiplied by the trap's stiffness gives the amplitude Δ*F* of oscillating load on the myosin from this bead in this event. Since the two beads oscillate in phase, the amplitude of oscillations in the total load on the myosin is the sum of amplitudes from each bead.

Similarly, we calculated the amplitude of oscillations of positions in the unbound state, using long time intervals before and after the binding event with the constraint that they did not overlap with other binding events. The duration of the binding event was then determined as ‘full width at half value' for the ‘peak' in Δ*x*(*t*) that a binding-event causes, over the background value of Δ*x*(*t*) in the unbound state (Fig. 1g–i). This procedure also gave *t*_1_ and *t*_2_. We applied it to both beads and, as a filter against false positives (fake brief events due to Brownian and other noise), we accepted only binding events having var(*t*_1_)+var(*t*_2_)<5(ms)^2^, where the variances are between the two beads. For accepted binding events, we averaged the two values for *t*_s_ from the two beads, and used that as the duration of the binding event.

We also calculated zero-frequency wavelet transforms *x*^(0)^(*t*) of the time series of bead positions in their respective traps. These are just sliding time averages of the positions such as shown in Fig. 1d–f, averaged over one period of the stage motion. We averaged these local averages over the duration of the attachment for noise reduction to get *x*^(0)^ (Fig. 1j–l). Multiplied by a trap's stiffness, such a mean position gives the mean load on the myosin from that trap. As the loads from the two traps oscillate in phase, the mean load (*F*_0_) on the myosin equals the sum of the mean loads from the two traps.

For illustration in the time domain, we fitted sine functions with period equal to the period of the stage motion to the binding events shown in Fig. 1j–l (red curves). We used the amplitude (Δ*x*), offset (*x*_0_) and phase as fitting parameters. Another sine function was fitted to the large time intervals with no binding before and after the binding event to model the bead excursions under hydrodynamic influence (orange curves in Fig. 1j–l). Such fits result in the same parameter values as we find using wavelet transforms. The advantage of wavelet transforms is that they tell us when attachment and detachment occur, that is, they deliver the instants in time, *t*_1_ and *t*_2_, at which we change fitting function in Fig. 1j–l.

### Temporal resolution

Our ability to detect brief attachment events is limited by the period of our oscillations of the stage, that is, 5 ms for 200 Hz. We needed at least one period, that is, 5 ms, to accurately determine parameters (*F*_0_, *ΔF* and *t*_s_). This temporal resolution was sufficient to detect the load-dependent ADP release rate of human β-cardiac myosin II. However, if one wants to explore the rates of even faster motors, such as human α-cardiac motor or skeletal muscle myosin II, for which, presumably, 0.1 ms time resolution is needed, then one needs a shorter period. The limiting factor on the frequency of oscillations in the experiments reported here is the piezoelectric stage, which is ∼500 Hz for our piezoelectric stage (PI). However, one can oscillate the dual traps with the acousto-optic deflectors that control their positions^29^, and in that manner oscillate at higher frequencies. The limiting factor for the time resolution is actually the compliance of the dumbbell: for the adiabatic description to hold, the period of oscillations should not approach the relaxation time of the dumbbell, which is half the relaxation time of the beads in their traps, in the present case ∼50 μs. Stronger traps give shorter relaxation time, but there is only so much tension that an actin filament and its attachments to beads will support without snapping. Smaller beads will also give the dumbbell shorter relaxation time, if the trapping force is not proportionally smaller. This is feasible, but it is very limited how much smaller beads can be and yet provide substantial tension in the dumbbell.

### Data analysis

For each of the seven molecules, we obtained a number of binding events (*N*=138, 388, 569, 174, 539, 950 and 229). Each binding event was characterized by three parameters, (*F*_0_, Δ*F*, and *t*_s_). For a given molecule, we partitioned these events into groups according to their *F*_0_-values, a group being those events with *F*_0_ in the same narrow interval, typically 1–2 pN wide, as exemplified by the red box in Fig. 2a. For each such group, its *t*_s_-values were binned into a histogram with bin width equal to one period of the stage oscillation, as exemplified in Fig. 2b. This binning avoids that the velocity and phase of the stage at the moment of attachment influences the statistics, and it ensures that the measured distribution of binding times is exponential (equation (5)). The first bin started at the cutoff of 1 stage period introduced above. To each such set of binned data, we fitted equation (5) using maximum likelihood estimation (red curve in Fig. 2b) to obtain *k*(〈*F*_0_〉, Δ*F*). The variance on this estimate was calculated as the inverse Fisher information for the parameter.

We then calculated the mean values 〈*F*_0_〉 in each bin and plotted 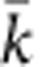 against that (Fig. 2c). We fitted equation (9) to that, using maximum likelihood estimation (red curve in Fig. 2c), to determine *k*_0_ and *δ*, while using 〈Δ*F*〉 instead of Δ*F* everywhere for a given molecule. Δ*F* is not entirely independent of *F*_0_ for a given molecule, but Δ*F* occurs only as an argument in *I*_0_ in equation (9), which varied sufficiently slowly as a function of the measured values of Δ*F* to justify the approximation.

The theoretical covariance matrix of the single-molecule estimates for *k*_0_ and *δ* was obtained as the inverse Fisher information matrix, from which we calculated the theoretical errors on the estimates under assumption that finite statistics was the only source of error (error bars on single-molecule values in Fig. 3a,b). These errors were used to calculate the weighted mean values across the molecules (blue lines, Fig. 3a,b). The seven single-molecule values scattered more than justified by their theoretical error bars alone, so we proceeded to calculate the s.e.m. directly from the seven measured values (blue regions, Fig. 3a,b), to realistically estimate the performance of the method.

### Actin pyrene labelling

Actin was labelled as described^50^. Briefly, F-actin was dialysed into 50 mM Hepes (pH 7.5), 100 mM KCl and 0.2 mM CaCl_2_ at 4 °C. F-actin was labelled with fivefold excess pyrene malemide overnight at 23 °C in the dark. The reaction was quenched with tenfold excess DTT, and undissolved pyrene was sedimented in a tabletop centrifuge at 11,000*g* for 15 min. F-pyrene-actin was then cycled into G-pyrene-actin at 4 °C by dialysing into G-buffer containing 2 mM Tris (pH 8), 0.2 mM ATP, 0.2 mM CaCl_2_ and 10 mM DTT. Any undepolymerized F-actin was precipitated at 100,000*g* for 30 min at 4 °C. G-pyrene-actin was then dialysed into 25 mM Hepes (pH 7.5), 25 mM KCl, 4 mM MgCl_2_, 0.2 mM CaCl_2_ and 10 mM DTT to make F-pyrene-actin and was used within a week before recycling the actin again from F-actin to G-actin. The concentration and percent labelling of G-actin was determined by the Bradford assay for protein concentration and the extinction coefficient of pyrene at 344 nm (22,000 M^−1^ cm^−1^)^51^. Before each experiment, an equimolar amount of unlabelled phalloidin was added to stabilize the F-pyrene-actin.

### ADP release rate measurement with a stopped-flow experiment

Solution phase ADP release rates for human β-cardiac S1 were measured by a HiTech SF-61DX2 (TgK Scientific Ltd, UK) stopped-flow apparatus at 23 °C. The buffer used in these experiments contained 25 mM Hepes (pH 7.5), 25 mM KCl, 4 mM MgCl_2_, 0.2 mM CaCl_2_ and 10 mM DTT. Prior to each experiment, human β-cardiac S1 was dialysed in the experimental buffer until the ratio of absorbance at 260 nm and 280 nm was ≤0.6. This ensured that most of the contaminant ATP was removed. Pyrene-labelled actin was then mixed with S1 and ADP to a final concentration of 1 μM F-pyrene-actin, 1 μM human β-cardiac S1 and 50 μM ADP. After an incubation of at least 5 min over ice, this mixture was rapidly mixed with buffer containing 4 mM ATP and 50 μM ADP in the stopped-flow instrument. Light (365 nm) was used to preferentially excite F-pyrene-actin. A 400-nm-long pass filter (Chroma Technology) was used to collect the fluorescence signal. For each different fresh preparation of myosin, ≥10 traces were collected and fit individually to a single exponential function as described below. Five such fresh myosin preparations were used in this study to obtain a mean ADP release rate of human β-cardiac S1.

### Stopped-flow data analysis

Fluorescent transients for the stopped-flow measurements were recorded at intervals of 100 μs over a 100-ms time window. The resulting 1,000 values (plotted as black symbols in Fig. 3c) were grouped into 40 different bins on the time axis with 25 values in each. The mean value in each bin (the bin average) is shown with red symbols in Fig. 3c. A three-parameter single exponential equation 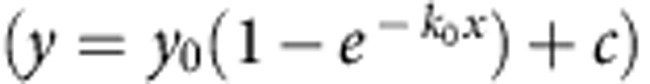 was fitted to the bin-averaged values using the least-squares method with equal weights to each bin. The fitted curve was used to detrend the variance of data in each bin. From each detrended variance, the s.e.m. of the corresponding bin average was calculated, using s.e.m.=√(variance/*N*). The inverse square of this s.e.m. was then used as weight factor for a second round of fitting of the same single exponential equation. The result of this fit was used for a better detrending of data in each bin, to obtain a better s.e.m. on each average, which was used for another round of weighted fitting followed by detrending, and so on repeatedly in a loop in our fitting program, until the parameter values of the fit did not change any more. The final fit is shown as a blue solid line in Fig. 3c. Its value for *k*_0_ is a measure of the unloaded ADP release rate of a single preparation of myosin. Five such different fresh preparations were used to report a mean±s.e.m. unloaded ADP release rate of human β-cardiac S1.

## Additional information

**How to cite this article:** Sung, J. *et al.* Harmonic force spectroscopy measures load-dependent kinetics of individual human β-cardiac myosin molecules. *Nat. Commun.* 6:7931 doi: 10.1038/ncomms8931 (2015).

## Supplementary Material

Supplementary Information Supplementary Figure 1, Supplementary Note 1 and Supplementary References
