# Supplementary material for: Harmonic force spectroscopy measures load-dependent kinetics of individual human β-cardiac myosin molecules
Source: Nat Commun. 2015 Aug 4;6:7931. doi: 10.1038/ncomms8931 (PMC4532873; doi:10.1038/ncomms8931)
Supplement: Supplementary Information — Supplementary Figure 1, Supplementary Note 1 and Supplementary References [file ncomms8931-s1.pdf]

Supplementary Figure 1

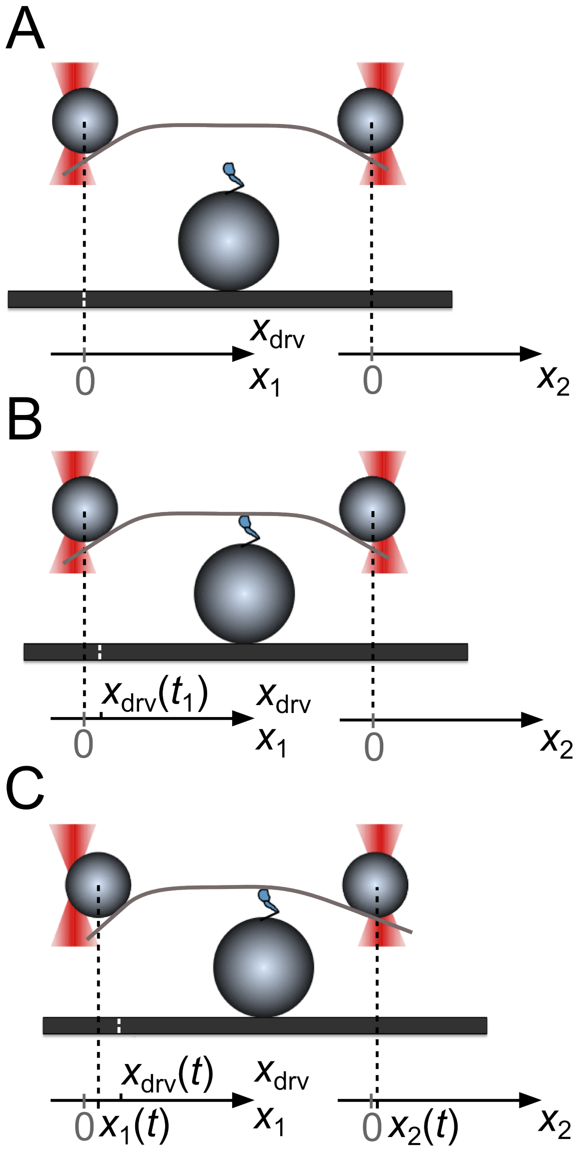

Supplementary Figure 1. Definitions of coordinates for beads and stage. (Continued next page.)

Supplementary Figure 1, cont'd. (A) The unattached dumbbell in its mean position in the traps holding and stretching it. The displacement of Bead 1 from this mean position is its lab-coordinate x_1. The displacements of Bead 2 from *its* mean position is *its* lab-coordinate x_2. The stage, with the larger platform bead with a myosin S1 fragment on top, is here shown in its mean position. The physical point on the stage which, averaged over stage oscillations, coincides with the mean position of Bead 1 (white mark on stage), is used to mark the position of the stage. Its displacement from its mean position is the coordinate x_{drv} of the stage in the lab, where "drv" is shorthand for "drive." **(B)** The moment t_1 when the myosin attaches to the dumbbell; in the case shown here, $x_{\mathrm{drv}}(t_1) > 0$ and increasing. Note that the scale of the x-axes is exaggerated in comparison with the separation between the dumbbell beads: These $1\,\mu$m-diameter beads are separated typically by 3 to $5\,\mu$m, while the amplitude A_{drv} of the harmonic translation of the stage is chosen typically to be 50 nm. Drawn to scale, this amplitude is little more than the width of the tick-marks on the x-axes, and $-A_{\mathrm{drv}} \leq x_{\mathrm{drv}}(t) \leq A_{\mathrm{drv}}$. The size of the motor fragment and the thickness of the actin filament are both even more exaggerated. The shape shown here of the bent actin is not physical, but an artists's rendition of the physical bending, which is maximal at the points of attachment of the beads. **(C)** Later, at $t > t_1$ and $x_{\mathrm{drv}}(t) > x_{\mathrm{drv}}(t_1)$, the stage has pulled the dumbbell with it, which makes Bead 1 pull back on the myosin with a positive load that, in the shown geometry, is numerically larger than the small negative load from Bead 2. Due to compliance in the left half of the dumbbell, $x_1(t) < x_{\mathrm{drv}}(t) - x_{\mathrm{drv}}(t_1)$.

Supplementary Note 1: Forces on a molecular motor attached to a harmonically oscillated compliant dumbbell

A: Motion of an unattached, compliant dumbbell

The next section presents a useful pedagogical toy, the rigid dumbbell. It does not represent our reality: Experimentally, we found that the dumbbell showed its compliance even in the unattached state. This observation is explained by the top panel in Supplementary Figure 1, which follows Fig. 5 in Ref. 1: The actin "handle" of the dumbbell is attached tangentially to the two dumbbell beads. Thus, the distance between the two dumbbell beads easily changes in response to changes in the force that stretches the dumbbell: It requires only one degree of change in the orientation of the actin filament at its point of attachment to a bead to allow the center of a 1 μm-diameter bead to displace itself 9 nm, which is seen as follows.

In a stretched dumbbell, the pull on the beads keeps them in close physical contact with the actin filament even if the link to the filament is flexible. The filament is tangential to both beads (Supplementary Figure 1), so 1° of change of direction of the filament at a point of contact, makes the radius of the bead to the point of contact rotate by 1° as well. Since the radius is $0.5\,\mu$m long, the center of the bead is displaced by $1^\circ/360^\circ \times 2\pi \times 500\,\mathrm{nm} = 9\,\mathrm{nm}$. This displacement takes place in the direction parallel to the filament's direction at its point of contact, so the displacement's component along the x-axis is shorter than 9 nm.

In the dumbbell's unattached, oscillating state, the force stretching the dumbbell may oscillate as the dumbbell is dragged back and forth in the two traps holding it by the oscillatory flow of fluid surrounding it. If the two traps are Hookean springs with identical stiffnesses and the two beads have identical drag coefficients, the separation between the beads will not oscillate while the dumbbell's position in the traps does. Our traps were not sufficiently identical to ensure constant separation, so our dumbbells displayed compliance even in their unattached states.

B: Motion of an unattached, rigid dumbbell

Consider the motion of an unattached, rigid dumbbell in response to the harmonic force on it caused by harmonic stage-oscillations. This simplest possible case of dumbbell motion illustrates aspects of the motion of an attached, compliant dumbbell. So it is a good starting point.

B-i: Definitions

Let x_1 and x_2 denote the lab-coordinates of Bead 1 and Bead 2, respectively, measured along the direction of the dumbbell and stage oscillation. Let the origin on the x_1-axis be the time-averaged position of Bead 1 in the unattached state of the dumbbell. With a similar definition of x_2 for Bead 2, the net trapping force on the dumbbell is zero when the beads are at these origins. In these considerations we leave out Brownian motion. It is easily added to Eq. (1) below, see Ref. 2, but by leaving it out, we arrive at trajectories that are already averaged with respect to Brownian motion, and hence ready for fitting to experimental trajectories.

Since the dumbbell is rigid, $x_1 = x_2$, and a single x-coordinate, $x_{\rm db}$, will describe its motion in the direction of its axis and the stage motion. We use $x_{\rm db} = x_1 = x_2$. The dumbbell is trapped with Hookean stiffness $\kappa_{\rm db} \approx \kappa_1 + \kappa_2$ and experiences a Stokes drag with coefficient $\gamma_{\rm db} \approx \gamma_1 + \gamma_2$, where κ_i and γ_i are, respectively, the stiffness of Trap i and Stokes drag coefficient of Bead i, $i = 1, 2$. Actually we expect $\gamma_{\rm db}$ to be larger than $\gamma_1 + \gamma_2$ because the actin "handle" of the dumbbell also contributes to the drag coefficient of the dumbbell.

B-ii: Equation of motion for unattached, rigid dumbbell

Newton's 2nd law with vanishing inertial term gives the equation of motion,

$$0 = -\kappa_{\rm db}\, x_{\rm db} - \gamma_{\rm db}(\dot{x}_{\rm db} - v_{\rm drv}) \ . \tag{1}$$

It is linear in $x_{\rm db}$ with an inhomogeneous source term proportional to the known stage-velocity, $v_{\rm drv}$, since this is also the velocity of the buffer fluid, which drives the motion of the dumbbell:

$$v_{\rm drv}(t) = A_{\rm drv}\, \omega_{\rm drv} \cos(\omega_{\rm drv} t) \ . \tag{2}$$

Here $A_{\rm drv}$ is the amplitude of the harmonic oscillation of the position of the stage, and $\omega_{\rm drv}$ is its cyclic frequency:

$$x_{\rm drv}(t) = A_{\rm drv} \sin(\omega_{\rm drv} t) \ . \tag{3}$$

We have chosen the origin of our time axis to be a point in time when $x_{\rm drv} = 0$ and $v_{\rm drv} > 0$. Thus, phase-angles of other oscillations are measured relatively to those of the stage position; $v_{\rm drv}$, e.g., is phase-shifted $\pi/2$ ahead of $x_{\rm drv}$.

B-iii: Solution $x_{\rm db}(t)$ to equation of motion

The dynamic equation (1) for $x_{\rm db}$ is solved (Sec. IIIB in Tolic-Nørrelykke *et al.*, 2006) by

$$\begin{aligned} x_{\rm db}(t) &= \frac{A_{\rm drv}}{[1+(f_{\rm c,db}/f_{\rm drv})^2]^{1/2}} \cos(2\pi f_{\rm drv}(t-\tau_{\rm db})) \\ &= \frac{v_{\rm drv}(t-\tau_{\rm db})}{[(2\pi f_{\rm c,db})^2+(2\pi f_{\rm drv})^2]^{1/2}} \\ &= \frac{x_{\rm drv}(t-\tau_{\rm db}+t_{\rm drv}/4)}{[1+(f_{\rm c,db}/f_{\rm drv})^2]^{1/2}} \ , \end{aligned} \tag{4}$$

where we have introduced the period $t_{\rm drv}$ of stage oscillations, the corner frequency $f_{\rm c,db}$ for the trapped dumbbell, and its inverse, the characteristic relaxation time, $\tau_{\rm db}$, for the dumbbell in the trap;

$$2\pi f_{\rm c,db} = \frac{\kappa_{\rm db}}{\gamma_{\rm db}} \quad ; \quad \tau_{\rm db} = \frac{1}{2\pi f_{\rm c,db}} = \frac{\gamma_{\rm db}}{\kappa_{\rm db}} \ . \tag{5}$$

B-iv: Typical parameter values; approximations to $x_{\rm db}(t)$

Typical values are $f_{\rm drv} = 200\,{\rm Hz}$ and $f_{\rm c,db} \approx 2\,{\rm kHz}$. So $(f_{\rm c,db}/f_{\rm drv})^2 \approx 100$. Consequently, we commit less than 1% error with the approximation

$$\begin{aligned} x_{\rm db}(t) &\approx \frac{v_{\rm drv}(t-\tau_{\rm db})}{2\pi f_{\rm c,db}} \\ &= \frac{f_{\rm drv}}{f_{\rm c,db}} x_{\rm drv}(t-\tau_{\rm db}+t_{\rm drv}/4) \ , \end{aligned} \tag{6}$$

which shows that the dumbbell oscillates with approximately 10% of the amplitude of the stage and trails the stage velocity by $\tau_{\rm db}$.

At $f_{\rm c,db} = 2\,$kHz, $\tau_{\rm db} = 80\,\mu$s, to be compared with the 5 ms period of the stage at 200 Hz. It shows that the dumbbell responds almost instantly to the drag force cause by the stage motion, with a delay of only 0.016 period, i.e., a phase angle of -0.1 radian or -6°. Thus, within a 2% error we can ignore $\tau_{\rm db}$ and have

$$\begin{aligned} x_{\rm db}(t) &\approx \frac{v_{\rm drv}(t)}{2\pi f_{\rm c,db}} \\ &= \frac{f_{\rm drv}}{f_{\rm c,db}} x_{\rm drv}(t + t_{\rm drv}/4) \end{aligned} \tag{7}$$

i.e., the dumbbell coordinate is phase shifted $\pi/2$, one fourth of a stage period, *ahead* of the stage coordinate. This phase shift ahead should not confuse, if one remembers that it is the stage *velocity* that drives the position of the unattached dumbbell away from zero, and this happens essentially in synchrony, with the dumbbell position trailing the stage velocity only by $\tau_{\rm db}$.

C: Motion of an attached, compliant dumbbell

C-i: Definitions

We neglect the compliance of the attachment of the dumbbell to the stage, i.e., the compliance in the S1, its linkage to the platform bead, and in the platform bead. We trust it to be negligible compared to the compliance in the dumbbell itself (Dupuis *et al.*, 1997). With this approximation, the point of attachment between dumbbell and S1 simply follows the stage. This approximation decouples the dynamics of the two parts of the dumbbell that connect at the attachment point. Consequently, we can treat those two parts independently. We start with the part including Bead 1.

Let t_1 denote the point in time when the S1 attaches to the dumbbell handle. For $t \leq t_1$, $x_1(t) = x_{\rm db}(t)$, where $x_{\rm db}(t)$ is given in Eq. (4) for the case of a rigid, unattached dumbbell. To keep things simple, we will proceed with this case, using $x_{\rm db}(t_1)$ as initial condition for the dynamics of $x_1(t)$ for $t > t_1$. It will disappear so fast from the description of the attached dumbbell that this choice does not matter. If we used the more correct initial condition, the coordinate $x_1(t_1)$ of a compliant, unattached dumbbell,

it would disappear equally fast from the description, so we don't bother to find it by solving for the motion of a compliant, unattached dumbbell.

The S1 attaches at a point on the dumbbell handle that we don't see and hence don't know. Since it isn't the midpoint, typically, Bead 1's and Bead 2's connection to the attachment-point have different compliances. For moderate relative movement of beads with respect to their attachment points, it is reasonable to assume that each compliance is well modeled by a Hookean spring. Let $\kappa_1^{(\mathrm{cp})}$ and $\kappa_2^{(\mathrm{cp})}$ denote the stiffnesses of these two springs.

C-ii: Equation of motion for bead in attached, compliant dumbbell

At the instant attachment occurs (Supplementary Figure 1, Panel B), our reference-point on the stage is located at $x_{\mathrm{drv}}(t_1)$, but it keeps moving, now with the dumbbell moving along with it. At a later time it is located at $x_{\mathrm{drv}}(t)$ (Supplementary Figure 1, Panel C). The difference, $x_{\mathrm{drv}}(t) - x_{\mathrm{drv}}(t_1)$, would, if the dumbbell were rigid, describe the displacement of every point on the dumbbell relatively to where it were at time t_1, when we have added to it the displacement δx of the dumbbell caused by the power stroke of the S1. The sign of δx depends on the direction of the power stroke.

At a later time t, Bead 1 would thus, if the dumbbell were rigid, have coordinate

$$\begin{aligned} x_1^{(\mathrm{rg})}(t) &= x_1(t_1) + x_{\mathrm{drv}}(t) - x_{\mathrm{drv}}(t_1) + \delta x \qquad (8) \\ &= A\left(\frac{\omega_{\mathrm{drv}}}{\omega_{\mathrm{c}}}\cos(\omega_{\mathrm{drv}}t_1) + \sin(\omega_{\mathrm{drv}}t) - \sin(\omega_{\mathrm{drv}}t_1)\right) + \delta x \ . \end{aligned}$$

The actual coordinate $x_1(t)$ of Bead 1 will differ from $x_1^{(\mathrm{rg})}(t)$ due to compliance, and this difference then causes a restoring Hookean force

$$F_1^{(\mathrm{cp})}(t) = -\kappa_1^{(\mathrm{cp})}\left(x_1(t) - x_1^{(\mathrm{rg})}(t)\right) \ . \qquad (9)$$

The difference between $x_1(t)$ and $x_1^{(\mathrm{rg})}(t)$ also causes a drag force on Bead 1: since $x_1(t) - x_1^{(\mathrm{rg})}(t)$ is not constant, the velocity in the lab of Bead 1, $dx_1/dt(t) = \dot{x}_1(t)$, generally differs from the velocity of the buffer fluid, which moves with the stage and $x_1^{(\mathrm{rg})}(t)$, and hence velocity $v_{\mathrm{drv}}(t)$. The ensuing drag force on Bead 1 equals $-\gamma_1(\dot{x}_1(t) - v_{\mathrm{drv}}(t))$.

Ignoring inertia, the total force on Bead 1 is zero at all times t according to Newton's 2nd law,

$$\begin{aligned} 0 &= -\kappa_1 x_1 + F_1^{(\mathrm{cp})} - \gamma_1(\dot{x}_1 - v_{\mathrm{drv}}) \\ &= -\kappa_1 x_1 - \kappa_1^{(\mathrm{cp})}\left(x_1 - x_1^{(\mathrm{rg})}\right) - \gamma_1\left(\dot{x}_1 - v_{\mathrm{drv}}\right) \\ &= -\gamma_1 \dot{x}_1 - (\kappa_1 + \kappa_1^{(\mathrm{cp})})x_1 + \kappa_1^{(\mathrm{cp})} x_1^{(\mathrm{rg})} + \gamma_1 v_{\mathrm{drv}} \ . \end{aligned} \quad (10)$$

This is the equation of motion for x_1. The equation of motion for x_2 is obtained by substituting subscript 1 for 2 in Eqs. (8–10).

C-iii: Physical meaning of terms in equation of motion for x_1

Imagine $x_1^{(\mathrm{rg})}(t)$ is positive and growing. This is the trajectory that Bead 1 would need to follow in order to follow the stage in unison. Imagine that it does not do that perfectly: Imagine $x_1(t)$ also is positive and growing, but trailing behind $x_1^{(\mathrm{rg})}(t)$, because it is held back by the trap to some extent, and the compliance of its connection with the stage allows it to yield somewhat to the force from the trap (Supplementary Figure 1, Panel C). Thus, the force from the trap is negative, while the drag force from the buffer fluid is positive. The sum of these two forces is transmitted through the compliant dumbbell to the S1. It is not quite the trapping force $-\kappa_1 x_1$ that we can measure by monitoring Bead 1's displacement x_1 in Trap 1. The force transmitted to the S1 is reduced in magnitude by the drag force, and equals $-\kappa_1 x_1 - \gamma_1\left(\dot{x}_1 - v_{\mathrm{drv}}\right)$.

The question now is how much the drag-force matters in the case of finite compliance. If it is negligible, the load on the myosin from Bead 1 equals the force that we can measure as $-\kappa_1 x_1$. To decide whether this simplification is reasonable, we solve the full problem below.

C-iv: How to recover the rigid dumbbell from the compliant dumbbell in the limit of no compliance

Equation (10) simplifies in the limit of zero compliance. This is the limit of $\kappa_1^{(\mathrm{cp})} \to \infty$. In that limit, x_1 differs infinitesimally from $x_1^{(\mathrm{rg})}$, just enough to make $-\kappa_1^{(\mathrm{cp})}\left(x_1 - x_1^{(\mathrm{rg})}\right) = \kappa_1 x_1$, while the drag-force term, $-\gamma_1\left(\dot{x}_1 - v_{\mathrm{drv}}\right)$, vanishes in that limit, because $x_1 \to x_1^{(\mathrm{rg})}$ means $\dot{x}_1 \to \dot{x}_1^{(rg)} = v_{\mathrm{drv}}$.

C-v: Solution to equation of motion for x_1

The solution to Eq. (10) for $t \geq t_1$ is

$$\begin{aligned} x_1(t) &= e^{-(t-t_1)/\tau_1} x_1(t_1) \\ &+ \int_{t_1}^{t} dt' e^{-(t-t')/\tau_1} \left(v_{\mathrm{drv}}(t') + \frac{\kappa_1^{(\mathrm{cp})}}{\gamma_1} x_1^{(\mathrm{rg})}(t') \right) \\ &= e^{-(t-t_1)/\tau_1} x_1(t_1) \\ &+ \int_{t_1}^{t} \frac{dt'}{\tau_1} e^{-(t-t')/\tau_1} \left(\frac{\kappa_1^{(\mathrm{cp})}}{\kappa_1 + \kappa_1^{(\mathrm{cp})}} x_1^{(\mathrm{rg})}(t') + \tau_1 \, v_{\mathrm{drv}}(t') \right) \end{aligned} \tag{11}$$

where we have introduced the characteristic time τ_1 for the relaxation of Bead 1 in the combined Hookean force fields from the optical trap and from the compliant section of the dumbbell between Bead 1 and the dumbbell's point of attachment to the stage,

$$\tau_1 = \frac{\gamma_1}{\kappa_1 + \kappa_1^{(\mathrm{cp})}} < \frac{\gamma_1}{\kappa_1} \approx \tau_{\mathrm{db}} \quad . \tag{12}$$

The first term on the right-hand side of Eq. (11) is a transient term that dies out exponentially fast in time with characteristic time τ_1. It represents the memory in $x_1(t)$ of its value at time t_1. The same exponentially decreasing "memory weight-factor" is seen also under the integrand in Eq. (11). It weighs the importance of past values (i.e., values at times before t) of the rest of the integrand for the value of $x_1(t)$. In that rest of the integrand, $x_1^{(\mathrm{rg})}(t')$ is the coordinate of the point on the stage that Bead 1 would follow if it followed the stage with rigor, i.e., with no compliance in the dumbbell, with $\kappa_1^{(\mathrm{cp})} = \infty$. Compliance reduces the amplitude of this contribution to the trajectory of $x_1(t)$ by the factor $\kappa_1^{(\mathrm{cp})}/(\kappa_1 + \kappa_1^{(\mathrm{cp})})$. The velocity v_{drv} in the integrand represents the velocity of the buffer fluid in the lab-frame of reference, but it is also the velocity of the stage, and hence of $x_1^{(\mathrm{rg})}$. This is used in the second of the following approximations to Eq. (11).

C-vi: Approximate solution revealing physics at play

We could insert the known explicit expressions for $x_1^{(\mathrm{rg})}(t)$ and $v_{\mathrm{drv}}(t)$ on the right-hand side of Eq. (11) and obtain the exact analytical expression of this equation of motion. That approach yields a complicated result. The

complications specify details on a time scale that we barely resolve, that of τ_1. Instead, we use the following simple approximation. It is precise because it keeps term to order $\tau_1/t_{\rm drv}$ in the description, and by doing only that it reveals the physics at play well: Consider the case of $\tau_1 \ll t_{\rm drv}$, i.e., the relaxation time τ_1 of the bead under the combined force of optical trap and dumbbell compliance is much shorter than the period of the stage oscillation. In this case, the functions $v_{\rm drv}(t')$ and $x_1^{\rm (rg)}(t')$ in the integrand in Eq. (11) change negligibly in the brief range of t'-values with $t' \leq t$ where the exponential weight-factor $\exp(-(t-t')/\tau_1)$ in Eq. (11) differs significantly from zero. We consequently can approximate $v_{\rm drv}(t')$ and $x_1^{\rm (rg)}(t')$ with their values in $t' = t - \tau_1$, the mean value of t' with respect to the weight-factor $\exp(-(t-t')/\tau_1)$. The same exponential factor makes the first term on the right-hand side of Eq. (11) negligible for $t - t_1 \gg \tau_1$. Taken together, these approximations give

$$\begin{aligned} x_1(t) &= \frac{\kappa_1^{\rm (cp)}}{\kappa_1 + \kappa_1^{\rm (cp)}}\, x_1^{\rm (rg)}(t-\tau_1) + \tau_1\, v_{\rm drv}(t-\tau_1) && (13)\\ &= \frac{\kappa_1^{\rm (cp)}}{\kappa_1 + \kappa_1^{\rm (cp)}} \left(x_1^{\rm (rg)}(t-\tau_1) + \frac{\gamma_1}{\kappa_1^{\rm (cp)}}\, v_{\rm drv}(t-\tau_1) \right) && (14)\\ &\approx \frac{\kappa_1^{\rm (cp)}}{\kappa_1 + \kappa_1^{\rm (cp)}}\, x_1^{\rm (rg)}\left(t - \tau_1 + \gamma_1/\kappa_1^{\rm (cp)} \right) \\ &= \frac{\kappa_1^{\rm (cp)}}{\kappa_1 + \kappa_1^{\rm (cp)}}\, x_1^{\rm (rg)}\left(t + \frac{\kappa_1}{\kappa_1^{\rm (cp)}}\tau_1 \right) \quad , && (15) \end{aligned}$$

where Eq. (15) involves yet an approximation. It uses that $v_{\rm drv}$ is the velocity of $x_1^{\rm (rg)}$, and is valid only for $\gamma_1/\kappa_1^{\rm (cp)} \ll t_{\rm drv}$. Equation (15) shows that Bead 1 on a compliant dumbbell oscillates with an amplitude that is a fraction of the stage amplitude, the fraction $\frac{\kappa_1^{\rm (cp)}}{\kappa_1+\kappa_1^{\rm (cp)}}$, and phase-shifted *ahead* of the stage position. It is ahead of the stage position, as far as phase is concerned, because it does not quite follow the stage as far as amplitude is concerned. This deficiency in amplitude causes a drag force on Bead 1 that pushes it in the direction the stage moves. It responds to this by shifting its position in the direction that the stage velocity points, which phase shifts the cycles of $x_1(t)$ towards the phase of $v_{\rm drv}$. This phase shift is positive. It is also always less than $\pi/2$, the phase of $v_{\rm drv}$ relatively to $x_{\rm drv}$. This is physically

obvious, and follows mathematically from Eq. (14) by inserting the explicit expressions for $x_1^{(\mathrm{rg})}(t)$ and $v_{\mathrm{drv}}(t)$ in it. Then it can be rewritten as

$$x_1(t) = x_1^{(0)} + \Delta x_1 \sin\left(\omega_{\mathrm{drv}}(t-\tau_1) + \phi_1\right) \tag{16}$$

where the cycle mean value $x_1^{(0)}$ is irrelevant for the phase, the phase shift $\phi_1 \in [0, \pi/2]$ is defined by

$$\tan\phi_1 = \frac{\gamma_1 \omega_{\mathrm{drv}}}{\kappa_1^{(\mathrm{cp})}} \ , \tag{17}$$

and the amplitude Δx_1 is

$$\Delta x_1 = \frac{\kappa_1^{(\mathrm{cp})}}{\kappa_1 + \kappa_1^{(\mathrm{cp})}} \frac{A_{\mathrm{drv}}}{\cos\phi_1} \ . \tag{18}$$

The phase shift of $x_1(t)$ in Eq. (16), $\phi_1 - \omega_{\mathrm{drv}}\tau_1$, is always positive for our parameter values, because its negative term is numerically small, $-\omega_{\mathrm{drv}}\tau_1 \approx -0.1$, while ϕ_1 is the arctan of a number strictly larger than that, though it also is small, typically.

C-vii: Load on S1 from Bead 1

The load $L_1(t)$ on S1 from Bead 1 equals minus the force on Bead 1 from trap and drag force. Thus the load is

$$L_1 = \kappa_1 x_1 + \gamma_1(\dot{x}_1 - v_{\mathrm{drv}}) = F_1^{(\mathrm{cp})} = -\kappa_1^{(\mathrm{cp})}\left(x_1 - x_1^{(\mathrm{rg})}\right) \tag{19}$$

where we have used Eq. (10). We find $x_1 - x_1^{(\mathrm{rg})}$ from Eq. (13) by using the very good approximation $x_1^{(\mathrm{rg})}(t-\tau_1) + \tau_1 v_{\mathrm{drv}}(t-\tau_1) \approx x_1^{(\mathrm{rg})}(t)$. With that

$$L_1(t) = -\kappa_1^{(\mathrm{cp})}\left(x_1(t) - x_1^{(\mathrm{rg})}(t)\right) = \frac{\kappa_1\kappa_1^{(\mathrm{cp})}}{\kappa_1 + \kappa_1^{(\mathrm{cp})}} x_1^{(\mathrm{rg})}(t-\tau_1) \ . \tag{20}$$

Note that both the cycle average and the amplitude of oscillations in this result remain unchanged if we entirely ignore the drag force. With $\gamma_1 = 0$, these two parameters remain the same in the approximation we have worked in here, which is a calculation up to and including the first order in τ_1/t_{drv}.

This load has a cycle-average

$$\langle L_1 \rangle = \kappa_1 x_1^{(0)} = \frac{\kappa_1 \kappa_1^{(\mathrm{cp})}}{\kappa_1 + \kappa_1^{(\mathrm{cp})}} \langle x_1^{(\mathrm{rg})} \rangle \tag{21}$$

and oscillates harmonically with amplitude

$$\Delta L_1 = \frac{\kappa_1 \kappa_1^{(\mathrm{cp})}}{\kappa_1 + \kappa_1^{(\mathrm{cp})}} A_{\mathrm{drv}} \ \ , \tag{22}$$

i.e., as if the only effect of compliance is to reduce the amplitude of oscillations by the factor $\kappa_1^{(\mathrm{cp})}/(\kappa_1 + \kappa_1^{(\mathrm{cp})})$.

The main effect of the drag force on Bead 1 in the attached, compliant dumbbell is to produce a small positive phase shift, $\phi_1 - \omega_{\mathrm{drv}}\tau_1$, in this bead's positions.

C-viii: Load on S1 from Bead 1 plus Bead 2

The load on S1 from Bead 2 is modeled like that from Bead 1. It oscillates with the same frequency as that from Bead 1, but typically with different amplitude and phase shift. The two phase shifts, $\phi_1 - \omega_{\mathrm{drv}}\tau_1$ and $\phi_2 - \omega_{\mathrm{drv}}\tau_2$, are both so small that even if they are not exactly the same, we can safely assume that the two loads, $L_1(t)$ and $L_2(t)$, are in phase. Consequently, the total load of the S1 from the dumbbell, $F(t) = L_1(t) + L_2(t)$, has a mean that equals the sum of means, and its amplitude equals the sum of amplitudes,

$$F_0 = \langle L_1 \rangle + \langle L_2 \rangle \ \ , \tag{23}$$

$$\Delta F = \Delta L_1 + \Delta L_2 \ \ , \tag{24}$$

which we have used after measuring $L_1(t)$ and $L_2(t)$. Not that it matters: If one wants to account for a difference between the phase-shifts of the two beads, one simply calculates F_0 and ΔF directly from $F(t) = L_1(t) + L_2(t)$ or includes the effect of the phase difference in Eqs. (23) and (24).

We conclude that we can treat the experiment as if only the trapping forces load the S1 and hence do this in phase with each other. We can ignore the drag force on the attached dumbbell because it mainly phase-shifts the load, without changing its mean value and its amplitude of oscillations.

Supplementary References

1. D. E. Dupuis, W. H. Guilford, J. Wu, and D. M. Warshaw, Actin filament mechanics in the laser trap. *J. Muscle Res. Cell Motil.* **18**, 17–30 (1997).

2. S. F. Tolić-Nørrelykke, *et al.*, Calibration of optical tweezers with positional detection in the back focal plane. *Rev. Sci. Instrum.* **77**, 103101 (2006).
